# Extract Derived From Black Rice Functions as a Photothermal Agent for Suppressing Tumor Growth and Metastasis

**DOI:** 10.3389/fbioe.2020.00904

**Published:** 2020-08-06

**Authors:** Muzhou Teng, Shuyi Zhou, Rongjun Zhang, Yu Zhang, Yang Xu, Xuemei Fu

**Affiliations:** ^1^School of Basic Medical Sciences, Southern Medical University, Guangzhou, China; ^2^The Eighth Affiliated Hospital, Sun Yat-sen University, Shenzhen, China

**Keywords:** black rice, photothermal therapy, tumor, lung metastasis, epithelial-mesenchymal transition

## Abstract

It remains a challenge to develop an effective therapeutic agent with low cost and good biocompatibility for cancer therapy. Based on its dark color, we hypothesized that, the extraction from black rice grains, denoted BRE, could serve as a photothermal conversion agent. The results showed that BRE confers a high photothermal conversion efficiency up to 54.13%. The combination of BRE and near infrared (NIR) treatment enables effective photothermal tumor ablation, and suppress tumor metastasis via inhibiting the epithelial-mesenchymal transition (EMT) pathway. In addition, BRE exhibits no obvious toxicity *in vivo*. Therefore, BRE could serve as a promising photothermal therapy agent with a low toxicity to treat cancer.

## Introduction

Despite great efforts that have been devoted to fight against cancer, it still poses a major threat to human health (Siegel et al., 2019). The current available strategies of cancer treatment include surgical therapy, chemotherapy (Yang et al., 2019), radiation therapy (RT) (Peng et al., 2020), photothermal therapy (PTT) (Jia et al., 2019), photodynamic therapy (PDT) (Wu et al., 2020), and immunotherapy (Feng et al., 2020). Among these cancer therapeutics, PTT has been developed vigorously in recent years which could convert the absorbed light energy into heat (Chen Y. et al., 2019). The advantage of PTT is that near infrared (NIR) could irradiate the subcutaneous and local tumor directly, so the constant high temperature could kill tumor cells precisely and ablate solid tumors(Chen D. et al., 2019). The black phosphorus (Chen et al., 2017), CuS–MnS_2_ nanomaterials (Chen W. et al., 2019), Au nanoparticle (Depciuch et al., 2020), and other metallic materials (Yuan et al., 2020) have been used as photothermal agent in cancer treatment. However, the metallic materials have certain drawbacks such as serious toxicity, expensive reagents, and complicated preparation procedure (Li et al., 2018), which have limited their further applications (Deng et al., 2019). Therefore, it is crucial to develop novel photothermal biomaterials with lower cost and better biocompatibility.

Compared with chemically synthetic biomaterials, natural biomaterials with excellent biocompatibility, and biodegradability are preferred in recent clinical trials. Currently, many biomaterials have been studied in photothermal ablation of tumors. For instance, natural sodium humate, biodegraded from the humic acid, had been applied as an excellent photothermal agents to induce tumor cell death (Miao et al., 2018). In addition, nanoparticles extracted from cuttlefish ink have been used to inhibit tumor growth by synergizing immunotherapy and PTT, showing excellent ability in the repolarization of tumor-associated macrophages and enhanced recruitment of cytotoxic T lymphocytes as well as photothermal killing effect (Deng et al., 2019). Inspired by studies to develop natural biomaterials, we are interested in developing cheaper and more accessible food-sourced PTT agents.

Black rice, mostly planted in the East Asia, is deemed a traditional and natural food (Samyor et al., 2017). It is widely used due to the low cost, easy accessible, and high nutritious value (Park et al., 2017), outstanding physicochemical characteristics and antioxidant potential (Pang et al., 2018). In addition, black rice is rich in water-soluble bioactive compounds such as phenolic acids, tocopherols, polyphenols, B vitamins, and anthocyanins (Wu et al., 2019) with excellent antioxidant, anti-obesity, and anti-diabetic capacity. These characteristics enabled black rice valuable for health and widely used as a food additive in the food processing industry (Aprodu et al., 2019). Anthocyanin, one of the major components of black rice, could significantly inhibit the proliferation, migration, and metastasis of breast cancer cells through targeting the RAS/RAF/MAPK pathway *in vitro* (Chen X.Y. et al., 2015). Moreover, black rice could enhance the immune response through inducing the proliferation and differentiation of immune cells *in vivo* (Fan et al., 2017). However, it remains unclear whether the black rice could be applied as a photothermal agent for tumor treatment.

The epithelial-mesenchymal transition (EMT) is curial for the metastatic behaviors of tumor cells (Hennessy et al., 2009). EMT involves a series of genetic and phenotypic changes, which contribute to the transformation of early epithelial cells into invasive malignant cancer cells (Lamouille et al., 2014). The process of EMT can be activated by genetic changes or other responses to external environment (Georgakopoulos-Soares et al., 2020). EMT also could promote early cancer cell to transdifferentiate into mesenchymal-like cells such as carcinoma cells and cancer stem cells, thus the cancer cell could acquire migration and invasion ability and detach from epithelial cell mass. During EMT process, the epithelial cells could lead to cell depolarization, reduced or even lost of cell–cell contacts and changing into a fibroblast-like morphology (Xu et al., 2018).

Developing therapeutic strategies with high efficiency as well as low toxicity and cost to drastically eliminate tumors is the ultimate goal in the cancer treatment. In this study, we have studied BRE as a photothermal agent for its high photothermal conversion efficiency up to 54.13%. This natural food could efficiently inhibit tumor growth and metastasis via EMT pathway with low toxicity. Prospectively, BRE might be a promising photothermal agent for tumor therapy.

## Materials and Methods

### Materials

BRE (containing 25% anthocyanin) was purchased from TIANXINGJIAN biochemical technology company (Xi An, China). Crystal violet was obtained from Beyotime biotechnology company (Shanghai, China). Fetal bovine serum (FBS), Phosphate buffer solution (PBS), Pyridine and dimethyl sulfoxide (DMSO), and Dulbecco’s modified eagle medium (DMEM) were provided by Gibco-BRL (Grand Island, New York, United States). LIVE/DEAD^TM^ Cell Imaging Kit was bought from Thermo Fisher Scientific (Waltham, MA, United States). Snail, vimentin, N-Cadherin, β-Actin antibody, radioimmunoprecipitation assay buffer (RIPA), and protease/phosphatase inhibitor cocktail (100×) were obtained from CST (Boston, MA, United States). 4% paraformaldehyde was obtained from Fude biotechnology company (Hangzhou, China). Matrigel was bought from Corning (Kangning, New York, United States).

### BRE Solutions Preparation

The concentration of the BRE solution was set as 20 mg/mL and stored in 4°C for further use.

### Cell Culture

Murine breast cancer cell 4T1 cell line (4T1 cells) was obtained from ATCC and cultured with DMEM containing 10% FBS and 1% penicillin-streptomycin solution (100×) at 37°C with 5% CO_2_ humidified atmosphere.

### The Detection of UV-vis-NIR Absorption Spectra

To explore the absorption spectra of BRE in the near infrared region (700–900 nm), 3 mL BRE solutions with concentrations of 1, 2, 5, 10, 15, and 20 mg/mL were determined using the UV-2600 spectrophotometer (Shimadzu Co., Japan).

### Evaluation of Photothermal Effect and Photostability

To measure the photothermal effect of BRE, a series concentration of 1, 2, 5, 10, 15, and 20 mg/mL BRE were irradiated (808 nm, 1 W/cm^2^) for 10 min, and the temperature change was recorded by an infrared thermal imaging camera (Shanghai Xilong Optoelectronics Technology Co. Ltd., China). Besides, the 10 mg/mL concentration of BRE was irradiated and recorded at different power density (0.5, 0.75, 1, and 2 W/cm^2^) for 10 min, respectively. To further study the photostability, the real-time temperature change of BRE by irradiating 10 mg/mL solution with an 808 nm laser (1 W/cm^2^) for 10 min (laser on) and then cooling to room temperature without irradiation for 10 min (laser off) were recorded. Such heating/cooling processes were repeated four times and used in the calculation of photothermal conversion efficiency. And the details of calculation were given in previous work (Hou et al., 2018).

### Live/Dead Staining Assay

4T1 cells were seeded on 96-well plates at a density of 5 × 10^3^ cells/well and incubated with DMEM complete growth medium for 24 h. Then, the cells were treated with different concentrations (0, 2, 5, and 10 mg/mL) of BRE for 24 h. After that, the cells were irradiated with an 808 nm laser (1 W/cm^2^) for 5 min and stained using the Live/Dead^TM^ Cell Imaging Kit for 30 min. Then, the cells were washed twice with PBS and the living cells or dead cells were observed and photographed using a fluorescent microscope (Nikon ECLIPSE Ti-U, Japan).

### Inhibition of Cloning Ability

4T1 cells were seeded on 6-well plates at a density of 2000 cells/well and incubated with DMEM medium for 24 h. Then, the cells were treated with different concentrations (0, 0.5, and 1 mg/mL) of BRE and were irradiated for 10 min (808 nm, 1 W/cm^2^), and were incubated for another 12 h and 24 h respectively. At fixed time points, the cells were washed twice with PBS and cultured with fresh DMEM medium for another 7 days. Then the cells were washed twice with PBS and fixed with 4% paraformaldehyde for 20 min. After that, the cells were washed twice with PBS and stained with 1% crystal violet dissolved in PBS for 30 min. The colony numbers were counted, and photographs were taken using a microscope (Nikon ECLIPSE Ti-U, Japan).

### *In vivo* Anti-tumor Effect

Four-week old female BALB/c mice were purchased from animal laboratory center of Guangdong Province and housed in SPF laboratory animal room. And all animal experiments were approved by Institutional Animal Care and Use Committee (IACUC). The mice were divided into four groups including control group, PBS + NIR, BRE and BRE + NIR group (*n* = 6). 1 × 10^6^ 4T1 cells resuspended in 100 μL PBS were subcutaneously injected into the flanks of mice. When tumor size reached 100 mm^3^, 50 μL of BRE solution (20 mg/mL) or PBS were intratumorally injected into the tumor region of the mice. The control group received no treatment and the BRE group was only intratumorally injected BRE solution. The mice of PBS + NIR treatment groups and BRE + NIR group were irradiated for 10 min (808 nm, 1 W/cm^2^). During irradiation, temperature change and thermal images of these mice were monitored by an infrared thermal imaging camera. After that, the relative tumor volume and body weight were recorded every two days. The survival rate was calculated when all mice were dead or the mice of control, PBS + NIR and BRE group were dead while the mice of BRE + NIR group remained alive 40 days after treatment. To examine the pathological changes of the tumor, one tumor-bearing mouse from each group was sacrificed one day after treatment, and the tumors were dissected and stained with H and E.

### Inhibition of Migration Ability

4T1 cells were suspended in serum-free DMEM medium at a density of 5 × 10^5^ cells (200 μL) and seeded in the upper chamber of the transwell. The lower cell chamber was added with 500 μL DMEM complete growth medium. Then, the cells were treated with different concentrations (0, 0.5, and 1 mg/mL) of BRE and were irradiated for 10 min (808 nm, 1 W/cm^2^). After being cultured for another 24 h, the upper cells were cleaned with cotton swab and transwells were washed twice with PBS and fixed with 4% paraformaldehyde for 20 min. And the cells were washed twice with PBS and stained with 1% crystal violet dissolved in PBS for 30 min. The membranes were obtained and fixed with neutral gum on the slides overnight. Then the slides were photographed using a microscope (Nikon ECLIPSE Ti-U, Japan).

### Inhibition of Invasion Ability

Matrigel (50 μL) was added into the upper chamber of the transwell insert and incubated at 37°C for 2 h. Then 4T1 cells were seeded on the transwell upper chamber at a density of 5 × 10^5^ cells/well (200 μL) and incubated with serum-free DMEM medium. The lower chambers were 24-well plates added with 500 μL DMEM complete growth medium. Later, the cells were treated with different concentrations (0, 0.5, and 1 mg/mL) of BRE and irradiated for 10 min (808 nm, 1 W/cm^2^). After 72 h incubation, the upper chambers were cleaned with cotton swab and the transwells were washed twice with PBS and fixed with 4% paraformaldehyde for 20 min. Then the cells were washed twice with PBS and stained with 1% crystal violet dissolved in PBS for 30 min. The membranes were obtained and fixed with neutral gum on the slides overnight. Pictures of the slides were taken using a microscope (Nikon ECLIPSE Ti-U, Japan).

### Western Blotting

Western blotting was used to determine the expression level of EMT related proteins. In brief, 4T1 cells were cultured in 6-well culture plates (3 × 10^5^ cells/well) overnight. The cells were treated with different concentrations (0, 0.5, and 1 mg/mL) of BRE and were irradiated for 10 min (808 nm, 1 W/cm^2^), the cells were incubated for another 24 h. Afterward, the cells were collected and whole-cell extracts were prepared with a RIPA buffer containing 1% protease/phosphatase inhibitor cocktail (100×), and separated by 10–12% sodium dodecyl sulfate polyacrylamide gel electrophoresis SDS-PAGE, and transferred to nitrocellulose membranes (Millipore). Antibodies including snail (3879s, CST), vimentin (5741s, CST), N-Cadherin (13116s, CST), β-actin (4967s, CST) were used in this study.

### Evaluation of *in vivo* Lung Metastasis Inhibition

Four-week old female BALB/c mice were divided into two groups including control group and BRE + NIR group. 1 × 10^6^ 4T1 cells resuspended in 100 μL PBS were subcutaneously injected into the flanks of mice. When the tumor size was reached 100 mm^3^, 50 μL of BRE solution (20 mg/mL) or PBS were intratumorally injected into the tumor region of the mice. The mice of control groups and BRE + NIR group were irradiated for 10 min (808 nm, 1 W/cm^2^). 30 days later, all mice were sacrificed and lungs were obtained and photographed alone. The numbers of lung metastasis were further counted, and lung slides were stained with H and E.

### Evaluation of *in vivo* Animal Toxicity

Four-week old female BALB/c mice were divided into two groups including control group and BRE treatment group. The mice of BRE treatment group were intravenously injected with 100 μL BRE solution (20 mg/mL) while the control group received 100 μL PBS solution in the same method. After that, the mice were housed for 30 days until they were sacrificed for toxicity assay. 150 μL whole blood were collected in tubes with spray-coated K_2_EDTA for future blood routine assessment including WBC, RBC, HGB, HCT, MCV, MCH, MCHC, and PLT assay. 500 μL whole blood were collected in normal EP tubes for future assessment of hepatic and renal toxicity. Blood samples for biochemical test were temporarily stored in -20°C for 2 h, and were incubated in 37°C for 1 h before centrifuged for 10 min (3000 rpm). After that the serum was collected for AST, ALT, BUN, and SCR assay. Major organs (including heart, liver, spleen, lung, and kidney) of all mice were collected after sacrifice. All major organs for histological analyses were fixed in 10% neutral buffered formalin, processed, and embedded in paraffin, cut into 4-μm-thick sections, and subsequently stained with H and E. H and E staining analysis were used to assess the *in vivo* toxicity.

### Statistical Analysis

Quantitative data were expressed as mean ± standard deviation (SD). The statistical differences were assessed using One-way ANOVA analysis. All tests were analyzed using statistical software (SPSS version 20.0). *P*-values of < 0.05 were considered to be statistically significant.

## Results and Discussion

### Characterization of BRE

**FIGURE 1:**
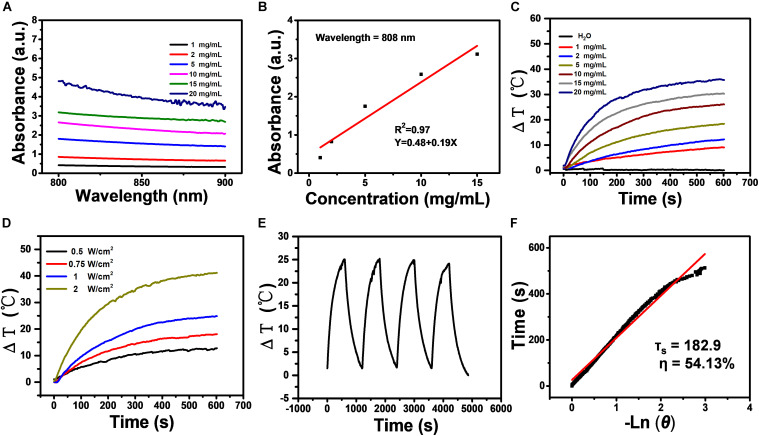
Characterization of BRE. **(A)** The vis-NIR absorbance spectrum (700–900 nm) of BRE with different concentrations. **(B)** The fitting curve of the absorbance of BRE at 808 nm with different concentrations (*R*^2^ = 0.97). **(C)** Photothermal images and corresponding temperature elevation of BRE with different concentrations under an 808 nm laser irradiation (10 min, 1 W/cm^2^). **(D)** Temperature elevation of BRE (10 mg/mL) with different power densities under an 808 nm laser irradiation. **(E)** Heating/cooling curves of BRE (10 mg/mL, 1 W/cm^2^) for four repeated irradiation cycles. **(F)** The fitting linear curve of time data versus -lnθ acquired from the cooling period, and the time constant (τ_s_) for heat transfer was calculated to be 182.9 s.

Exacted from black rice containing 25% anthocyanin, was derived by serial dilution of BRE powder purchased from TIANXINGJIAN Biochemical Technology Company with PBS (pH = 7.4). First, the vis-NIR absorbance spectrum (700–900 nm) of BRE solutions was assessed. The absorbance of BRE solutions was smoothly decreased from 700 nm to 900 nm, and the relation with different concentrations was close to linear with the *R*^2^ up to 0.97 at 808 nm laser (Figures 1A,B), indicating that BRE solutions had a strong absorbance in NIR wavelength. When irradiated for 10 min at 808 nm (1 W/cm^2^), the temperature of BRE solution was increased faster and higher with the increasing concentrations of BRE solutions. As a negative control, the same irradiation had little impact on PBS solution. The ΔT of the BRE solution at the concentration of 20 mg/mL was about 35.2°C, indicating the photothermal capability that matches many photothermal materials such as Pd@Au/Ce6/PAH/H-MnO_2_ (Figure 1C; Liu et al., 2020). In addition, we further showed that the photothermal performance of BRE was dependent on the irradiation power density (Figure 1D). The ΔT of the BRE solutions irradiated with the power density at 2 W/cm^2^ was about 41.3°C. As shown in Figure 1E, the temperature of BRE solutions (10 mg/mL) was increased sharply when the laser was on for 10 min (1 W/cm^2^) and decreased when the laser was off. The temperature change was highly reproducible similarly to many photothermal materials such as FA-CuS/DTX@PEI-PpIX-CpG nanocomposites (Chen L. et al., 2019), suggesting the superb photothermal stability of BRE solutions.

Based on the above data, the photothermal conversion efficiency (η) of BRE solutions was calculated according to the previously reported method (Hou et al., 2018). The fitting linear curve of time data versus -lnθ was acquired from the cooling period, and the time constant (τ_s_) for heat transfer was calculated to be 182.9 s. The η value of BRE solution was determined to be 54.13% (Figure 1F), which was obviously superior to commercial gold nanorods (21%) (Hessel et al., 2011). In summary, these results indicated that the BRE solution is a promising candidate for PTT.

### Evaluation of *in vitro* Anti-tumor Effect

**FIGURE 2:**
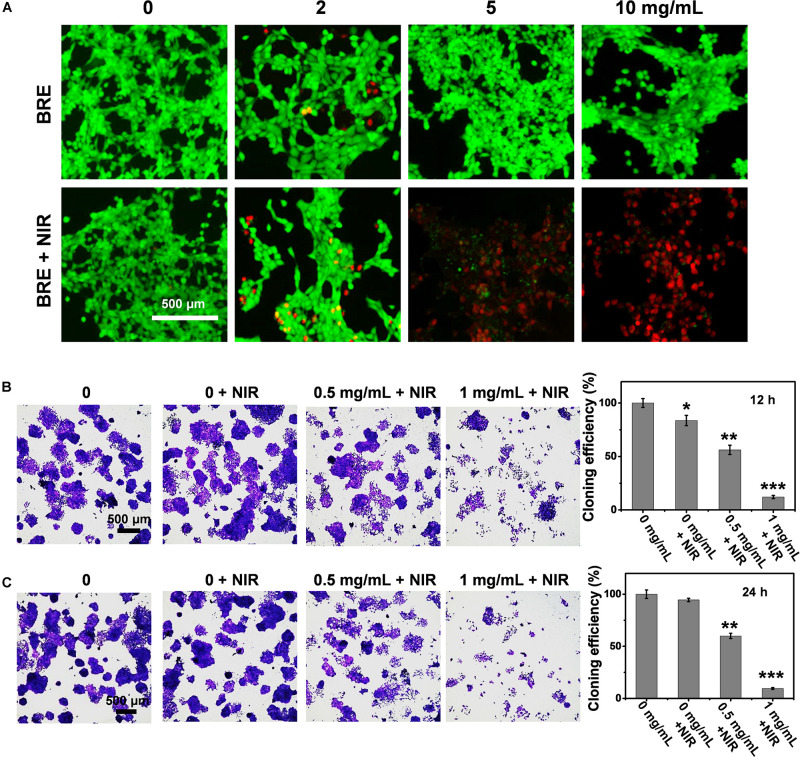
The evaluation of *in vitro* anti-tumor effect. **(A)** The Live/Dead fluorescence images of 4T1 cells treated with BRE at different concentrations for 24 h and placed under an 808 nm laser irradiation (1 W/cm^2^) for 5 min. **(B)** Photograph of 4T1 clone forming, quantification of 4T1 cloning efficiency with the BRE and NIR treatment in 12 h. **(C)** Photograph of 4T1 clone forming, quantification of 4T1 cloning efficiency with the BRE and NIR treatment in 24 h. *P < 0.05, **P < 0.01, ***P < 0.001 compared with the control.

To investigate the photothermal tumor cell killing ability of BRE *in vitro*, Live/Dead staining assay was conducted. While the cells were alive in the BRE group, there was massive cell death in the culture after the treatment with NIR (Figure 2A). In further support of the photothermal killing ability of BRE, the colony formation ability of tumor cells after BRE + NIR treatment was assessed. The cloning formation efficiency of tumor cells at 12 and 24 h after BRE (1 mg/mL) + NIR treatment was significantly reduced (Figures 2B,C). Therefore, the combinational treatment of tumor cells with BRE and NIR have apparent anti-tumor activity *in vitro*.

### Evaluation of *in vivo* Anti-tumor Effect of BRE

**FIGURE 3:**
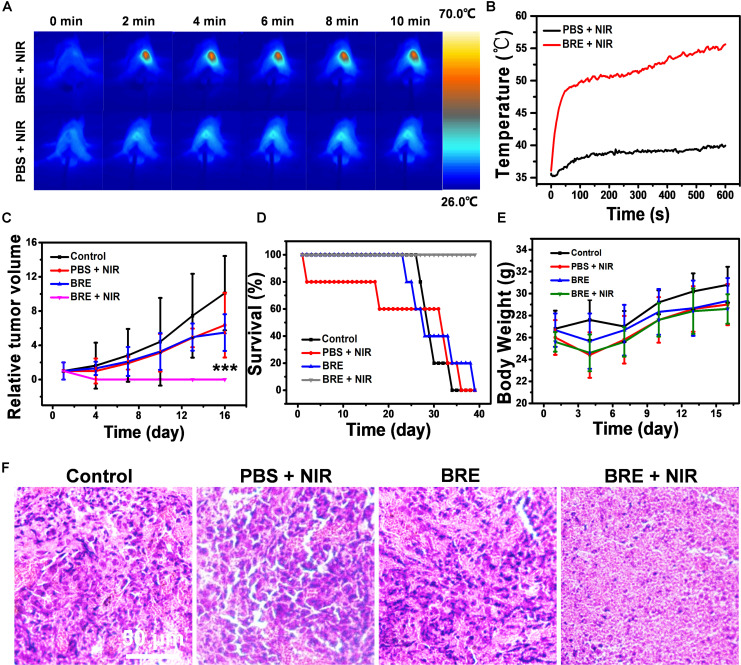
The evaluation of *in vivo* anti-tumor effect. **(A)** Photothermal images and **(B)** temperature curves of BALB/c mice bearing 4T1 tumors irradiated with 808 nm for 10 min (1 W/cm^2^). **(C)** The relative tumor volume of different treatment groups. **(D)** The survival rate of different treatment groups. **(E)** The body weight of different treatment groups. **(F)** The tumor HE staining of different treatment groups. Data are presented as mean ± SD. ***P < 0.001.

To evaluate the *in vivo* anti-tumor effect, BALB/c mice harboring syngeneic 4T1 tumors were divided into four groups including control, PBS + NIR, BRE, and BRE + NIR group (*n* = 6). During BRE + NIR treatment, the local temperature of 4T1 tumors was increased to 55.4°C, much higher than that of PBS + NIR group (29.1°C), enough to kill tumor cells and ablate subcutaneous tumors *in vivo* (Figures 3A,B). In addition, the size of the tumors after BRE + NIR treatment was smaller than that after other three treatments, suggesting that the combinational BRE and NIR treatment could effectively suppress tumors *in vivo* (Figure 3C). The survival rate of tumor-bearing mice after BRE + NIR treatment was much higher than that of other three treatments (Figure 3D). The body weight of tumor-bearing mice after various treatments showed no apparent difference, suggesting that BRE + NIR treatment confers a low toxicity (Figure 3E). Histological analysis of the tumors showed that the tumors after BRE + NIR treatment exhibited extensive necrosis, indicating efficient tumor ablation (Figure 3F). These results indicated that the combinational BRE and NIR treatment could efficiently cause local hyperthermia of tumor tissues, and thus presenting a promising treatment for solid tumors with superb killing efficiency.

### Mechanisms of *in vitro* and *in vivo* Suppression of Lung Metastasis

**FIGURE 4:**
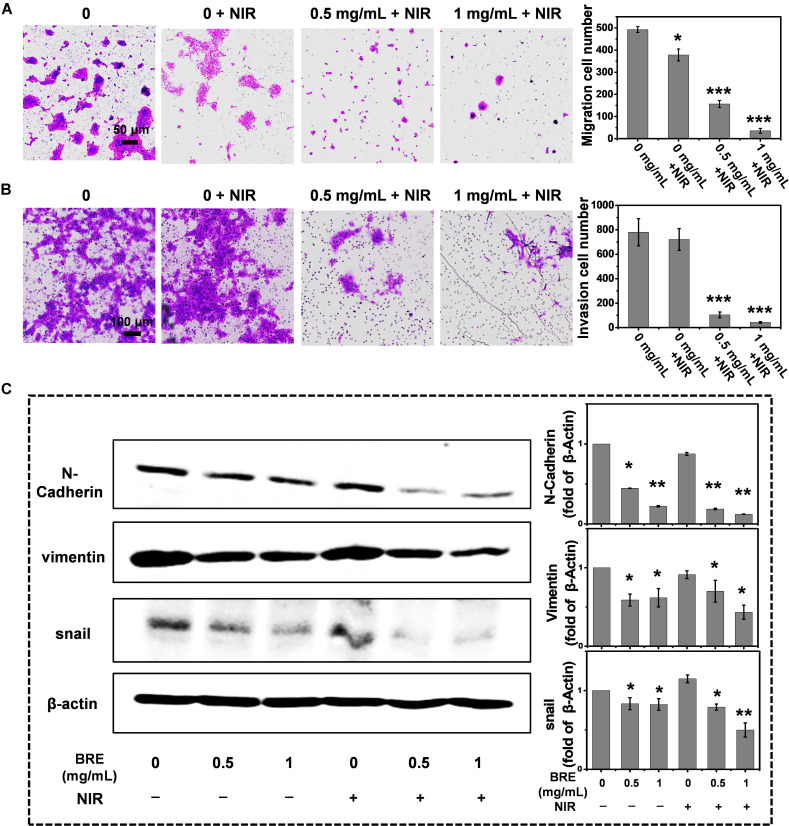
Inhibition of migration, invasion and anti-EMT ability. **(A)** Photograph and quantification of 4T1 migration ability with the BRE and NIR treatment. **(B)** Photograph and quantification of 4T1 invasion ability with the BRE and NIR treatment. **(C)** Western blotting of relative mesenchymal markers including N-cadherin, Vimentin and Snail. Data are presented as mean ± SD. *P < 0.05, **P < 0.01, ***P < 0.001 compared with the control.

Preventing tumor recurrence or inhibiting tumor metastases is as important as the ablation of original tumors (Jin et al., 2018). Therefore, the impact of BRE + NIR treatment on migrational ability and invasive ability was also studied. The migration and invasion of tumor cells were significantly reduced after the BRE (1 mg/mL) + NIR treatment when compared to other three treatments (Figures 4A,B).

Because EMT is required for the metastatic behaviors of invasion and migration, we examined the expression of EMT related mesenchymal markers, including N-cadherin, Vimentin, and Snail (Polyak and Weinberg, 2009). Our data showed that BRE treatment reduced the expression of Snail, Vimentin, and N-Cadherin, indicating that BRE treatment exhibits strong anti-EMT effects. In addition, NIR enhanced the anti-EMT effectiveness, suggesting that the combinational BRE and NIR treatment inhibits the transformation of early epithelial cells into mesenchymal stem cells (Figure 4C).

**FIGURE 5:**
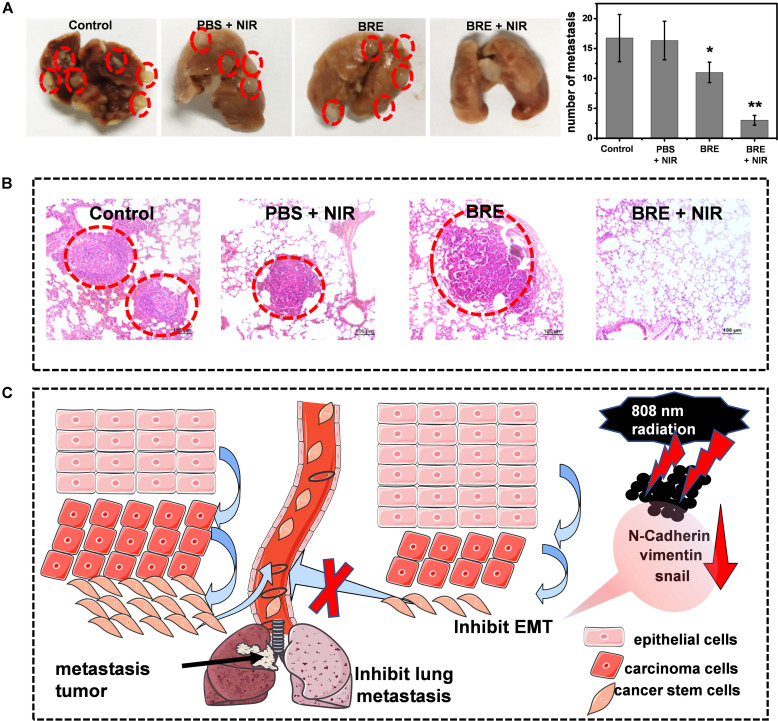
Mechanisms of *in vivo* inhibiting lung metastasis. **(A)** Photographs and numbers of lung metastases in different treatment groups. **(B)** The HE staining of lung in different treatment groups. **(C)** Schematic diagram of BRE and laser radiation for inhibiting lung metastasis and EMT. Data are presented as mean ± SD. *P < 0.05, **P < 0.01 compared with the control.

Epithelial-mesenchymal transition could contribute to the dissociation of cancer cells from the primary tumor foci and intravasation into blood vessels (Hennessy et al., 2009) leading to distant metastases in other organs (Obenauf and Massagué, 2015). In order to assess the inhibitory ability of BRE + NIR on lung metastasis *in vivo*, lung metastasis model was conducted. Our data indicate that only BRE and BRE + NIR treatments suppressed the lung metastasis (Figures 5A,B), In addition, BRE + NIR treatment was more potent than BRE treatment in suppressing lung metastasis and improving survival (Figures 5A,B). Therefore, PTT and anti-EMT activities of BRE + NIR treatment contribute to inhibiting the formation of metastatic lung nodules (Figure 5C; Chen W. et al., 2015).

### Evaluation of *in vivo* Toxicity

**FIGURE 6:**
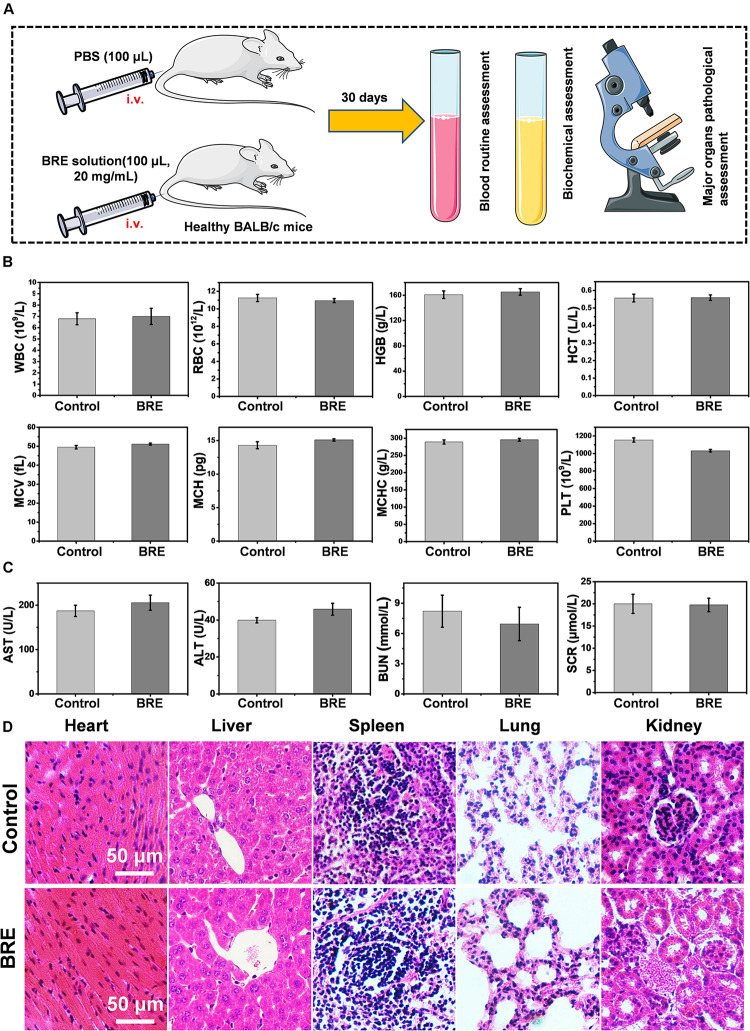
The evaluation of *in vivo* toxicity. **(A)** The scheme of *in vivo* toxicity assay. **(B)** The blood routine assessment of control and the BRE (20 mg/mL) treatment group. **(C)** Physiological function assessment of hepatic and renal toxicity from control and BRE (20 mg/mL) treatment group. **(D)** The HE staining of major organs of control and BRE (20 mg/mL) treatment group. Data are presented as mean ± SD.

To evaluate the *in vivo* toxicity of BRE, the BALB/c mice were injected intravenously with 100 μL BRE solution (20 mg/mL) while the control group received the same volume of PBS solution. 30 days after treatment, all mice were sacrificed for *in vivo* toxicity study. The blood was subjected to the blood routine assessment, hepatic, and renal toxicity assessment. Besides, the HE staining of major organs were also analyzed to detect the toxicity of BRE (Figure 6A). Blood routine assessment included WBC, RBC, HGB, HCT, MCV, MCH, MCHC, and PLT assays, the indexes showed little difference between the control and BRE treatment group, supporting the notion that BRE have no apparent blood toxicity *in vivo* (Figure 6B). Moreover, *in vivo* hepatic and renal toxicity was evaluated by AST, ALT, BUN, and SCR assays respectively. Our results showed that these values were identical between the control and BRE group, indicating that BRE did not induce hepatic and renal toxicity (Figure 6C). In addition, the histological analysis indicated that the major organs of BRE treated mice showed little pathological changes, suggesting that BRE did not induce systemic toxicity (Figure 6D). Together, these data show that BRE does not induce blood toxicity, hepatic and renal toxicity or even systemic toxicity *in vivo*, and thus representing a safe photothermal agent for *in vivo* application.

## Conclusion

In summary, BRE, the exaction of the traditional food black rice, has been developed as a novel and low-cost PTT agent with effective anti-tumor and anti-metastasis abilities. With excellent photothermal stability and photothermal conversion efficiency (54.13%), the temperature of BRE could be increased high enough to induce tumor cell death. In the context of anti-tumor and anti-metastasis activities, the results show that the combination of BRE and NIR treatment could significantly inhibit the tumor growth by elevating the local hyperthermia and metastasis by suppressing EMT. In addition, the *in vivo* toxicity data show that BRE causes no obvious systemic toxicity and paved the way for its clinical use. In summary, promising therapeutic effectiveness, low cost, and low toxicity highlight the potential of BRE in tumor therapy.

## Data Availability Statement

All datasets presented in this study are included in the article/Supplementary Material.

## Ethics Statement

The animal study was reviewed and approved by IACUC of Southern Medical University.

## Author Contributions

YX and XF conceived of the original research idea. MT, SZ, and RZ performed the experiments and analyzed the data. YZ helped with the animal experiments. YX, XF, MT, SZ, and RZ prepared the manuscript. All authors contributed to the article and approved the submitted version.

## Conflict of Interest

The authors declare that the research was conducted in the absence of any commercial or financial relationships that could be construed as a potential conflict of interest.

## References

[B1] Aprodu I. Milea ŞA. Anghel R.-M. Enachi E. Barbu V. Crãciunescu O. (2019). New functional ingredients based on microencapsulation of aqueous anthocyanin-rich extracts derived from black rice (*Oryza sativa* L.). *Mol. (Basel. Switzerland)* 24:3389. 10.3390/molecules24183389 31540422 PMC6766832

[B2] Chen D. Tang Y. Zhu J. Zhang J. Song X. Wang W. (2019). Photothermal-pH-hypoxia responsive multifunctional nanoplatform for cancer photo-chemo therapy with negligible skin phototoxicity. *Biomaterials* 221:119422. 10.1016/j.biomaterials.2019.119422 31437723

[B3] Chen L. Zhou L. Wang C. Han Y. Lu Y. Liu J. (2019). Tumor-targeted drug and CpG delivery system for phototherapy and docetaxel-enhanced immunotherapy with polarization toward m1-type macrophages on triple negative breast cancers. *Adv. Mater.* 31:1904997. 10.1002/adma.201904997 31721331

[B4] Chen W. Wang X. Zhao B. Zhang R. Xie Z. He Y. (2019). CuS–MnS2 nano-flowers for magnetic resonance imaging guided photothermal/photodynamic therapy of ovarian cancer through necroptosis. *Nanoscale* 11 12983–12989. 10.1039/c9nr03114f 31264665

[B5] Chen Y. Li L. Chen W. Chen H. Yin J. (2019). Near-infrared small molecular fluorescent dyes for photothermal therapy. *Chinese Chem. Lett.* 30 1353–1360. 10.1016/j.cclet.2019.02.003

[B6] Chen W. Cao G. Yuan X. Zhang X. Zhang Q. Zhu Y. (2015). Notch-1 knockdown suppresses proliferation, migration and metastasis of salivary adenoid cystic carcinoma cells. *J. Trans. Med.* 13 167–167. 10.1186/s12967-015-0520-2 PMC4445799 25990317

[B7] Chen X.-Y. Zhou J. Luo L.-P. Han B. Li F. Chen J.-Y. (2015). Black rice anthocyanins suppress metastasis of breast cancer cells by targeting RAS/RAF/MAPK pathway. *BioMed Res. Int.* 2015 414250–41 4250. 26649302 10.1155/2015/414250 PMC4663286

[B8] Chen W. Ouyang J. Liu H. Chen M. Zeng K. Sheng J. (2017). Black phosphorus nanosheet-based drug delivery system for synergistic photodynamic/photothermal/chemotherapy of cancer. *Adv. Mater.* 29:1603864. 10.1002/adma.201603864 27882622

[B9] Deng R.-H. Zou M.-Z. Zheng D. Peng S.-Y. Liu W. Bai X.-F. (2019). Nanoparticles from cuttlefish ink inhibit tumor growth by synergizing immunotherapy and photothermal therapy. *ACS Nano* 13 8618–8629. 10.1021/acsnano.9b02993 31246413

[B10] Depciuch J. Stec M. Kandler M. Baran J. Parlinska-Wojtan M. (2020). From spherical to bone-shaped gold nanoparticles—Time factor in the formation of Au NPs, their optical and photothermal properties. *Photodiag. Photody. Ther.* 30:101670. 10.1016/j.pdpdt.2020.101670 31988022

[B11] Fan M.-J. Yeh P.-H. Lin J.-P. Huang A.-C. Lien J.-C. Lin H.-Y. (2017). Anthocyanins from black rice (Oryza sativa) promote immune responses in leukemia through enhancing phagocytosis of macrophages in vivo. *Exp. Therap. Med.* 14 59–64. 10.3892/etm.2017.4467 28672893 PMC5488472

[B12] Feng B. Niu Z. Hou B. Zhou L. Li Y. Yu H. (2020). Enhancing triple negative breast cancer immunotherapy by ICG-templated self-assembly of paclitaxel nanoparticles. *Adv. Funct. Mater.* 30:1906605 10.1002/adfm.201906605

[B13] Georgakopoulos-Soares I. Chartoumpekis D. V. Kyriazopoulou V. Zaravinos A. (2020). EMT factors and metabolic pathways in cancer. *Front. Oncol.* 10:499–499. 32318352 10.3389/fonc.2020.00499 PMC7154126

[B14] Hennessy B. T. Gonzalez-Angulo A.-M. Stemke-Hale K. Gilcrease M. Z. Krishnamurthy S. Lee J.-S. (2009). Characterization of a naturally occurring breast cancer subset enriched in epithelial-to-mesenchymal transition and stem cell characteristics. *Cancer Res.* 69 4116–4124. 10.1158/0008-5472.can-08-3441 19435916 PMC2737191

[B15] Hessel C. M. Pattani V. P. Rasch M. Panthani M. G. Koo B. Tunnell J. W. (2011). Copper selenide nanocrystals for photothermal therapy. *Nano Lett.* 11 2560–2566. 10.1021/nl201400z 21553924 PMC3111000

[B16] Hou M. Yan C. Chen Z. Zhao Q. Yuan M. Xu Y. (2018). Multifunctional NIR-responsive poly(vinylpyrrolidone)-Cu-Sb-S nanotheranostic agent for photoacoustic imaging and photothermal/photodynamic therapy. *Acta Biomaterialia* 74 334–343. 10.1016/j.actbio.2018.05.011 29753138

[B17] Jia H.-R. Zhu Y.-X. Liu X. Pan G.-Y. Gao G. Sun W. (2019). Construction of dually responsive nanotransformers with nanosphere–nanofiber–nanosphere transition for overcoming the size paradox of anticancer nanodrugs. *ACS Nano* 13 11781–11792. 10.1021/acsnano.9b05749 31553562

[B18] Jin L. Han B. Siegel E. Cui Y. Giuliano A. Cui X. (2018). Breast cancer lung metastasis: molecular biology and therapeutic implications. *Cancer Biol. Ther.* 19 858–868. 10.1080/15384047.2018.1456599 29580128 PMC6300341

[B19] Lamouille S. Xu J. Derynck R. (2014). Molecular mechanisms of epithelial-mesenchymal transition. Nature reviews. *Mol. Cell Biol.* 15 178–196. 10.1038/nrm3758 24556840 PMC4240281

[B20] Li J. Chen Z. Huang R. Miao Z. Cai L. Du Q. (2018). Toxicity assessment and histopathological analysis of nano-ZnO against marine fish (Mugilogobius chulae) embryos. *J. Environ. Sci.* 73 78–88. 10.1016/j.jes.2018.01.015 30290874

[B21] Liu Y. Li F. Guo Z. Xiao Y. Zhang Y. Sun X. (2020). Silver nanoparticle-embedded hydrogel as a photothermal platform for combating bacterial infections. *Chem. Eng. J.* 382:122990 10.1016/j.cej.2019.122990

[B22] Miao Z.-H. Li K. Liu P.-Y. Li Z. Yang H. Zhao Q. (2018). Natural humic-acid-based phototheranostic agent. *Adv. Healthcare Mater.* 7:1701202. 10.1002/adhm.201701202 29334186

[B23] Obenauf A. C. Massagué J. (2015). Surviving at a distance: organ-specific metastasis. *Trends Cancer* 1 76–91. 10.1016/j.trecan.2015.07.009 28741564 PMC4673677

[B24] Pang Y. Ahmed S. Xu Y. Beta T. Zhu Z. Shao Y. (2018). Bound phenolic compounds and antioxidant properties of whole grain and bran of white, red and black rice. *Food Chem.* 240 212–221. 10.1016/j.foodchem.2017.07.095 28946264

[B25] Park S.-Y. Lee J.-W. Kim G.-W. Kim H.-Y. (2017). Effect of black rice powder on the quality properties of pork patties. *Korean J. Food Sci. Anim. Resour.* 37 71–78. 10.5851/kosfa.2017.37.1.71 28316473 PMC5355586

[B26] Peng C. Liang Y. Chen Y. Qian X. Luo W. Chen S. (2020). Hollow mesoporous tantalum oxide based nanospheres for triple sensitization of radiotherapy. *ACS Appl. Mater. Interf.* 12 5520–5530. 10.1021/acsami.9b20053 31891473

[B27] Polyak K. Weinberg R. A. (2009). Transitions between epithelial and mesenchymal states: acquisition of malignant and stem cell traits. *Nat. Rev. Cancer* 9 265–273. 10.1038/nrc2620 19262571

[B28] Samyor D. Das A. B. Deka S. C. (2017). Pigmented rice a potential source of bioactive compounds: a review. *Int. J. Food Sci. Technol.* 52 1073–1081. 10.1111/ijfs.13378

[B29] Siegel R. L. Miller K. D. Jemal A. (2019). Cancer statistics, 2019. *Cancer J. Clin.* 69 7–34. 10.3322/caac.21551 30620402

[B30] Wu C. Sun J. Zheng P. Kang X. Chen M. Li Y. (2019). Preparation of an intelligent film based on chitosan/oxidized chitin nanocrystals incorporating black rice bran anthocyanins for seafood spoilage monitoring. *Carbohyd. Polym.* 222:115006. 10.1016/j.carbpol.2019.115006 31320067

[B31] Wu R. Wang H. Hai L. Wang T. Hou M. He D. (2020). A photosensitizer-loaded zinc oxide-polydopamine core-shell nanotherapeutic agent for photodynamic and photothermal synergistic therapy of cancer cells. *Chinese Chem. Lett.* 31 189–192. 10.1016/j.cclet.2019.05.004

[B32] Xu F. Li S. Zhang J. Wang L. Wu X. Wang J. (2018). Cancer stemness, immune cells, and epithelial–mesenchymal transition cooperatively predict prognosis in colorectal carcinoma. *Clin. Color. Cancer* 17 e579–e592. 10.1016/j.clcc.2018.05.007 29921496

[B33] Yang S. Zhou L. Su Y. Zhang R. Dong C.-M. (2019). One-pot photoreduction to prepare NIR-absorbing plasmonic gold nanoparticles tethered by amphiphilic polypeptide copolymer for synergistic photothermal-chemotherapy. *Chinese Chem. Lett.* 30 187–191. 10.1016/j.cclet.2018.02.015

[B34] Yuan M. Xu S. Zhang Q. Zhao B. Feng B. Ji K. (2020). Bicompatible porous Co3O4 nanoplates with intrinsic tumor metastasis inhibition for multimodal imaging and DNA damage–mediated tumor synergetic photothermal/photodynamic therapy. *Chem. Eng. J.* 394:124874 10.1016/j.cej.2020.124874

